# Keep the nest clean: survival advantages of corpse removal in ants

**DOI:** 10.1098/rsbl.2014.0306

**Published:** 2014-07

**Authors:** Lise Diez, Philippe Lejeune, Claire Detrain

**Affiliations:** 1 Unit of Social Ecology, Université Libre de Bruxelles, Bruxelles, Belgium; 2 Unité de Gestion des Ressources forestières et des Milieux Naturels, Université de Liège, Gembloux, Belgium

**Keywords:** necrophoresis, *Myrmica rubra*, social immunity, survival, ants

## Abstract

Sociality increases exposure to pathogens. Therefore, social insects have developed a wide range of behavioural defences, known as ‘social immunity’. However, the benefits of these behaviours in terms of colony survival have been scarcely investigated. We tested the survival advantage of prophylaxis, i.e. corpse removal, in ants. Over 50 days, we compared the survival of ants in colonies that were free to remove corpses with those that were restricted in their corpse removal. From Day 8 onwards, the survival of adult workers was significantly higher in colonies that were allowed to remove corpses normally. Overall, larvae survived better than adults, but were slightly affected by the presence of corpses in the nest. When removal was restricted, ants removed as many corpses as they could and moved the remaining corpses away from brood, typically to the nest corners. These results show the importance of nest maintenance and prophylactic behaviour in social insects.

## 1. Introduction

The social contacts that come with group living increase the risk of pathogen transmission, particularly for social insects, with their high density of genetically related individuals [1]. Consequently, social insects are known to defend themselves against disease outbreaks with a set of socially performed prophylactic behaviours known as ‘social immunity’. Recent findings have shown how diverse and sophisticated these behaviours are, ranging from pathogen avoidance while nesting, grooming with application of antimicrobial compounds and incorporation of antimicrobial material in the nest (reviewed in [2]). Social insects also use specific behaviours to avoid horizontal transmission of pathogens. First, diseased individuals may leave the nest on their own before they die [3,4] or be removed by nest-mates [5]. Termite workers isolate dead individuals by burying them [6], whereas honeybees and many ant species transport them outside the colony [7,8].

Despite the identification of several behaviours associated with social immunity, their effectiveness in terms of colony survival has been poorly investigated. A limited number of studies have shown the fitness gain due to hygienic behaviours (i.e. allogrooming) after a colony is challenged with a pathogen [9–12]. In this study, we aimed to investigate the benefits of social immunity in a situation where there is no artificial introduction of a pathogen to the colony. We tested whether corpse removal improves worker and brood survival in the common red ant, *Myrmica rubra*.

## 2. Material and methods

*Myrmica rubra* colonies were collected in Gembloux, Belgium. Ants were kept in plaster nests (Janet type, 85 × 85 × 2 mm) connected to foraging arenas (135 × 185 × 50 mm). The nest entrance consisted in a 15 mm hole perforated in the middle of the glass roof. Each nest contained no queens, 170–230 workers and 58–60 larvae. Laboratory conditions were kept at 23 ± 1°C and 45 ± 5% HR, with a constant photoperiod of 12 h d^−1^. Nest humidity was maintained by adding 75 ml of water three times a week in the two ditches surrounding the nest. Each colony was provided with ad libitum water and a modified Bhatkar diet containing 2 : 1 sugar/protein [13].

### (a) Experimental protocol

In order to quantify the influence of necrophoresis on ants, we compared the survival of *M. rubra* ants in colonies that were limited in their ability to remove corpses (limited removal: LR colonies, *N* = 15) to that of control colonies which were able to remove them normally (free removal: FR colonies, *N* = 15). Experiments were performed during two periods, in 2010 and 2012 (respectively, *N* = 7 and *N* = 8 for each treatment).

To hamper the ability of colonies to remove corpses, we covered the entrance with a 20 × 20 × 20 mm Plexiglas cube, which was perforated with 12 holes of 2 mm diameter each. The small holes permitted only one ant to pass at a time and made it hard to carry corpses. FR colonies were free to remove corpses through cubes with one big hole (15 mm diameter). Preliminary experiments on six colonies for each treatment showed that the type of nest entrance did not influence ant survival over a 52 day period (Cox model, *z* = 0.31, *p* = 0.76).

At the beginning of the experiment (Day 1), 10 corpses were placed in each nest. Corpses were nest-mates killed by freezing for 35 min at −24°C and left at room temperature for 3 h before their introduction into the nest. We counted the number of live and dead ants as well as the number of larvae twice a week for seven weeks. We also took a picture of the nest in order to localize corpses and the brood patch so that we could calculate the relative distance of corpses to larvae. This was defined as the distance from a corpse to the edge of the nearest larvae patch divided by the farthest possible distance from the edges of all larvae patches. We used specific software called Formi-GIS (details in [14]).

### (b) Data analysis

To test overall differences of survival between the two treatments, we used a Cox proportional hazards regression model, incorporating both colony and experimental period as random factors. To test differences in colony's survival between treatments for each day separately, we used Wilcoxon rank sum tests. When testing differences in the localization of corpses in the nest over time, we performed generalized linear mixed models (GLMM) applied to binomial distributions (logistic transformation), taking into account the colony as a random factor. If not otherwise specified, all means are given with standard deviations. All raw data are provided as .xls files in the electronic supplementary material. We used software R v. 3.0.1 (http://www.r-project.org) for all statistical analyses.

## 3. Results

### (a) Effect of necrophoresis on colony survival

**Figure 1.**
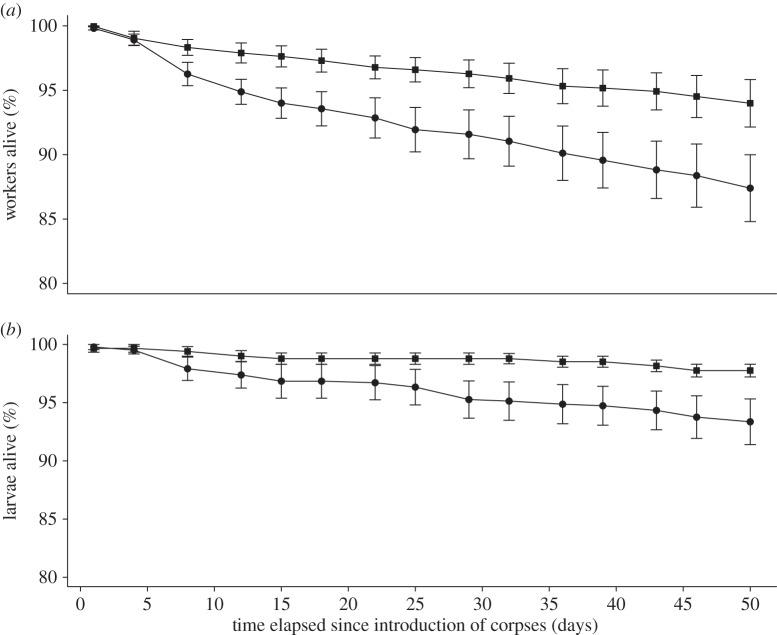
(*a*) Survival curves of workers (mean ± s.e.). (*b*) Survival curves of larvae (mean ± s.e.). Squares, FR colonies; circles, LR colonies.

We quantified the impact of corpse removal on the demography of ant colonies. Overall, workers in LR colonies survived less than those in FR colonies (Cox model, *z* = 13.5, *p* < 0.0001). At the end of the experiment, 87.4 ± 10.1% (*N* = 15) of workers in LR colonies and 94.0 ± 7.1% (*N* = 15) of workers in FR colonies were still alive (figure 1*a*). In order to understand at what time these differences became significant, we compared the survival rate day by day, finding workers' survival in FR colonies was higher than worker's survival in LR colonies from Day 8 onwards (Wilcoxon rank sum test, from Day 8 to Day 50, *W* ≥ 162, *N* = 15, *p* < 0.05).

The survival rate of larvae was always above that of workers in both conditions (figure 1*b*). Yet, we observed significant differences in the survival curves of larvae between the LR and FR colonies (Cox model, *z* = 5.34, *p* < 0.0001). At 50 days following the introduction of corpses into the nest, 93.4 ± 7.6% (*N* = 15) of larvae in LR colonies and 97.8 ± 2.1% (*N* = 15) of larvae in FR colonies were still alive. However, we observed no significant differences in the survival rates of LR and FR colonies while comparing each day separately (Wilcoxon rank sum test, from Day 1 to Day 50, *W* ≥ 84.5, *N* = 15, *p* < 0.05).

### (b) Location of corpses inside the nest

**Figure 2.**
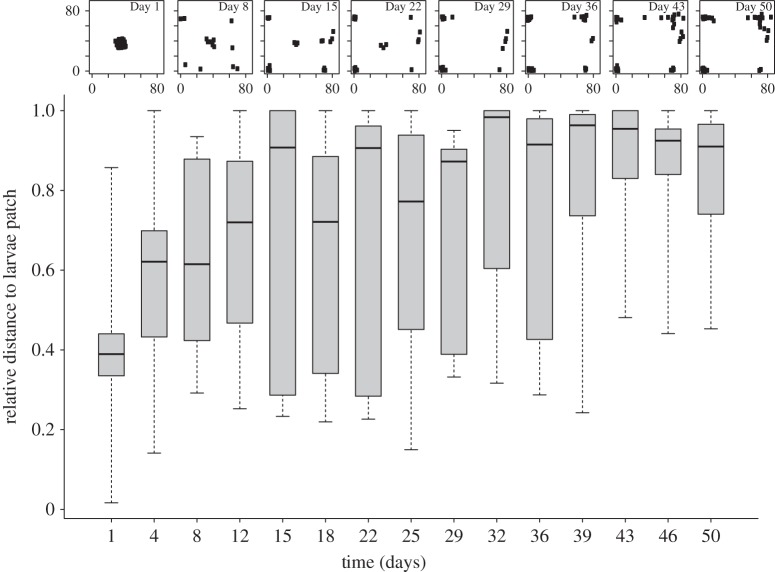
Boxplots represent the relative distance of corpses from the larvae patch. Above each pair of boxplots is a miniature of the nest (85 × 85 mm) on a given day after the introduction of corpses, where each small black dot represents one corpse (data from all colonies are pooled).

In FR colonies, corpses were removed rapidly, and none remained in the nest after 4 days. In LR colonies, most corpses remained in the nest until the fourth day. After 8 days, workers managed to cut some corpses into pieces and thus succeeded in removing these body parts out of the ‘small-holes’ nest entrance. Some colonies ultimately removed all corpses though on average 3.7 ± 2.6 (*N* = 91) corpses remained inside the nest. In this latter case, corpses were gradually moved away from brood patches (GLMM, *χ*^2^ = 85.1, d.f. = 1, *p* < 0.001). From Day 4, no corpses were in contact with larvae. From Day 12, most corpses were located in the most remote areas of the nest (often corners), with a relative distance to larvae of more than 0.75 (figure 2).

## 4. Discussion

We have shown that prophylaxis through corpse removal enhances ant survival, even if no additional pathogen load is introduced to the nest. Corpse removal has been widely assumed to provide fitness advantages for social insects, and theoretical simulations of pathogen transmission throughout colonies demonstrates this [15]; however, our study provides the first experimental evidence. Along with other prophylactic behaviours, corpse removal acts as a first line of defence against horizontal transmission of pathogens, thus allowing reduced investment in costly personal immune defences [16]. Indeed, in wood ants, the incorporation of antibacterial resin into the nest leads to a decrease in antibacterial and lytic activities in worker haemolymph [17]. Honeybees, which have a wide range of social immune responses, appear to have fewer selection constraints on genes related to individual immunity compared with non-social insects [18]. In our study, we actually observe the survival gain of keeping the nest clean from corpses. Corpses artificially staying longer in the nest may have increased the occurrence of microorganisms, requiring a greater investment in the immune system for live ants and possibly resulting in a reduced lifespan. Further research could test for the development of microorganism on corpses in the nest, and the consequences on the immune system activity, investigating the costs and benefits of necrophoresis.

In our experiments, larvae survived better than workers and the impact on brood survival in colonies with restricted corpse removal was smaller than for workers. This was not necessarily expected given larvae can be infected by all parasite taxa and are particularly susceptible while their body cuticle and gut lining are not fully developed [1]. However, we observed worker behaviours that reduced contact between brood and potential sources of pathogens. First, corpses were moved to more remote areas of the nest and were rarely in contact with larvae patches. Second, in *M. rubra*, younger workers are mainly involved in brood care, while workers involved in corpse transport mainly stay outside the nest or near the nest entrance [14]. Such spatial and behavioural compartmentalization is part of social immunity [2] and seems to promote larval survival.

After 8 days, workers in LR colonies managed to eject six to seven corpses from the nest despite the difficulties in doing so. Other corpses were taken to nest corners, or in some cases ‘buried’ using cotton wool extracted from water dispensers. Ants were nevertheless less prompt to show these alternate behaviours than they are to eject corpses out of the nest, which occurs within 24 h [19]. Corpse-burying is the main way used by termites to segregate corpses from the colony [6]. In ants, *Temnothorax lichtensteini* workers tend to naturally bury corpses in the nest [20], and in some attine species, workers dispose of waste and dead ants in dedicated chambers [21]. In *M. rubra*, corpse-burying might be an alternative to removal, which can be used in specific circumstances, such as winter when ants stay within the nest, and which may be sufficient in cases of low parasite pressure. Indeed, even when corpses remain in the nest, mortality rates are low for workers and larvae. Further research could evaluate behavioural modulation given different levels of parasitism.

## Supplementary Material

Preleminary experiments data

## Supplementary Material

Survival data

## Supplementary Material

Location data
